# Civic ecosystem responses to compound crises: evidence from Syria

**DOI:** 10.1111/disa.70070

**Published:** 2026-09-21

**Authors:** Munzer Alkhalil, Zedoun Alzoubi, Mazen Gharibah, Zaki Mehchy, Iavor Rangelov, Marika Theros, Rim Turkmani

**Affiliations:** ^1^ LSE IDEAS, The London School of Economics and Political Science, and David Nott Foundation United Kingdom; ^2^ LSE IDEAS, The London School of Economics and Political Science, United Kingdom, and Research for Health System Strengthening in Syria, Union of Medical Care and Relief Organizations Syria; ^3^ LSE IDEAS, The London School of Economics and Political Science United Kingdom

**Keywords:** civic ecosystems, complementarity, complex adaptive systems, compound crises, crisis response, Syria

## Abstract

Compound crises have attracted growing interest from scholars and policymakers in recent years. Little attention, however, has been directed to the social relations that emerge from and respond to such phenomena. This paper closes the gap by investigating the emergence, impact, and limitations of the civic ecosystem that responded to the earthquakes in northwest Syria in 2023, an area already affected by protracted armed conflict, mass displacement, and humanitarian emergency. We combine ideas from complex adaptive systems theory, civic ecosystems scholarship, and the literature on compound crises to analyse primary data and evidence collected through key informant interviews, a focus group discussion, and participatory observation. The paper contends that compound crises present distinctive challenges and opportunities that civic ecosystems are particularly adept at addressing and exploiting. We show how they evolve through adaptive cycles of learning and innovation across multiple co‐occurring shocks. One implication of the argument is the need to apply systems theory to understand how systems can tackle, rather than only produce, complex social problems such as compound crises.

## 1 INTRODUCTION

Compound crises involving co‐occurring shocks, disasters, and emergencies have attracted growing attention from scholars and practitioners in recent years. There is an established scholarship on compound and cascading risks (Wahl et al., 2015; Pescaroli and Alexander, 2018). Recent studies conceptualise compound crises as a unique type of problem in particular contexts such as urban settings (Westman et al., 2022), highlighting how the prevalence of compounding and interrelated crises requires new approaches to preparedness (Kruczkiewicz et al., 2021). Policymakers have focused on the interactions between multiple shocks and threats that trigger compound crises whereby the cumulative impacts are far greater than the sum of the individual components (Intini, Abdel‐Shafi, and Poole, 2020; Matanó et al., 2022). Yet, the question of what patterns of social relations emerge from and respond to compound crises has been largely neglected.

We seek to close this gap in the literature by investigating the role of civic ecosystems in addressing compound crises. The paper draws on ideas from complex adaptive systems (CAS) theory and the civic ecosystems framework (CEF) and uses evidence from the initial response to the February 2023 earthquakes in northern Syria, an area already affected by protracted armed conflict, mass displacement, and humanitarian emergency. It examines the emergence of a civic ecosystem for crisis response in that part of the country, assesses its impact, and considers its limitations.

We contend that compound crises present distinctive challenges and opportunities that civic ecosystems are particularly adept at addressing and exploiting. Because they evolve through adaptive cycles of learning and innovation across multiple co‐occurring events, they can respond to abrupt developments and shift some of the constraints and barriers generated by protracted co‐occurring crises. One implication of the argument is the need to apply systems theory to understand how systems can tackle, rather than only produce, complex social problems such as compound crises.

CAS theory explains the behaviour of complex systems by focusing on the dynamic interactions between the individual components of a system and between the system and its environment. It emphasises the ‘adaptive’ character of complex systems, which means their capability to learn and modify their behaviour (Ahmed, Elgazzar, and Hegazi, 2005; Rammel, Stagl, and Wilfing, 2007). CAS provides a theoretical foundation for CEF, which is an analytical tool for comprehending how patterns of social relations emerge organically to address pressing social problems. The concept of civic ecosystems brings out the distinctive logic and characteristics of this type of complex adaptive system (Rangelov and Theros, 2023). Combining these ideas provides a useful analytical lens for investigating the role of civic ecosystems in responding to compound crises.

The compound crises in northern Syria constitute a compelling case for applying and refining that analytical lens. More than a decade of armed conflict led to the fragmentation of governance structures, the displacement of millions of people, and severe damage to critical infrastructure, leaving opposition‐controlled areas in northern Syria with limited capacity to supply essential services. Economic collapse, ongoing hostilities, and the COVID‐19 (coronavirus disease 2019) pandemic further strained the region's already fragile healthcare system, while international sanctions and long‐standing restrictions imposed by the Syrian government created obstacles for humanitarian assistance delivery. When two earthquakes struck northern Syria on 6 February 2023, measuring 7.8 and 7.5 in magnitude, they amplified the persistent vulnerabilities created by these overlapping phenomena and revealed how, in compound crises settings, an acute shock interacts with pre‐existing protracted crises and adaptive behaviours.

The earthquakes devastated vital infrastructure, including homes, hospitals, and roads, overwhelming available resources in the immediate aftermath when international cross‐border aid was effectively blocked. The initial response was driven by local civic actors, including civil defence teams, health directorates, non‐governmental organisations (NGOs), and community networks and initiatives, while the Syrian diaspora mobilised to provide vital financial support and other assistance. A civic ecosystem emerged to respond to the abrupt shock of the disaster, which, as noted, augmented the region's multiple co‐occurring crises.

The paper makes several contributions to the literature. It advances nascent CEF scholarship and the growing body of work on compound crises by engaging their ideas and refining them with insights from empirical research to develop an analytical framework for studying the role of civic ecosystems in compound crises settings. It contributes to the CAS literature by shifting the focus from systems that generate a set of problems to systems that generate a set of social relations that address problems. The study also provides insights for practitioners and policymakers by highlighting the potential of civic ecosystems and how they may be leveraged in addressing compound crises. In doing so, it also contributes to localisation debates in humanitarian action by making visible locally embedded resources and networks that are created across multiple crises and can be activated when further disruption occurs.

The paper proceeds as follows. The next section discusses key concepts and insights from CAS theory, the literature on compound crises, and CEF scholarship, which are central to the analysis. The subsequent section elaborates the background and manifestations of the compound crises affecting northern Syria at the time of the earthquakes. This is followed by a note on the methodology. We then discuss the emergence, impact, and limitations of the civic ecosystem that carried out the bulk of the response in the immediate aftermath of the earthquakes. The final section draws out the key conclusions and implications of our findings for scholars, practitioners, and policymakers.

## 2 COMPLEX ADAPTIVE SYSTEMS, COMPOUND CRISES, AND CIVIC ECOSYSTEMS

CAS theory is a variation of systems theory that emphasises complexity and adaptation. CAS are defined by their ‘complex behaviour that emerges as a result of interactions among system components (or agents) and among system components (or agents) and the environment. Through interacting with and learning from its environment, a complex adaptive system modifies its behaviour to adapt to changes in its environment’ (Rammel, Stagl, and Wilfing, 2007, p. 10). The ability of the system to be dynamically influenced by or adapt to changes that emerge from inside and outside the system, sometimes referred to as ‘panarchy’, is a central characteristic of CAS (Coetzee, Van Niekerk, and Raju, 2016). Panarchy, however, is more than adaptability. It is best understood as a framework of cross‐scale interactions and nested adaptive cycles that explains how systems grow, collapse, and renew through dynamic feedback (Holling, 2001; Gunderson and Holling, 2002).

Other key characteristics emphasised in the CAS literature include emergence, non‐linearity, feedback loops, and context‐based responses (Innes and Booher, 1999; Railsback, 2001; Turner and Gardner, 2015). Emergence refers to the notion that system‐level properties and patterns emerge from micro‐level interactions between individual elements of the system, which generate characteristics that are different in kind. Non‐linearity is the idea that the size of inputs in the system is not proportionate to the outputs, whereby minor inputs might change the system in fundamental ways, whereas major inputs may not. Feedback loops are mechanisms through which learning and adaptation occur in the system in response to its dynamic environment. Context shapes the development and functioning of CAS in crucial ways and accounts for the uniqueness of each system. However, the context of a specific system is not static and can also change as a result of interactions between system agents or components. The CAS literature further draws attention to the importance of initial conditions and considers the role of path dependency in shaping system dynamics (Levin, 1998).

Growing interest in compound crises as a distinctive phenomenon reflects the idea of a world defined by polycrisis, where multiple shocks and disruptions co‐exist, interact, and amplify one another. The co‐occurrence of multiple crises has focused attention on the need to understand and address their compounded and interlinked impacts. The COVID‐19 pandemic, in particular, has spurred scholarship and policy analysis on such interconnections and triggered calls for rethinking the tendency to treat crises as singular and tackle them in isolation (Phillips et al., 2020; Au et al., 2022). The literature on compound crises builds on scholarship that distinguishes between different types of risk, such as compound, interconnected, interacting, and cascading risks, and considers their complementarity to support further research and policymaking (Pescaroli and Alexander, 2018; Zscheischler et al., 2018). In fragile and conflict‐affected contexts, policymakers have directed attention to the distinctive challenges arising in situations where multiple hazards occur simultaneously, creating feedback loops that amplify pre‐existing vulnerabilities and create future ones (Matanó et al., 2022).

Recent scholarship on compound crises draws on the CAS literature to illuminate their logics and characteristics. In urban settings, scholars have combined insights from CAS and critical urban studies in developing the concept of compound urban crises (Westman et al., 2022). They argue that CAS is a particularly useful analytical lens for understanding the systemic nature of compound crises. The CAS literature helps to capture how disruptions of governance and everyday life in compound crises reflect the interconnected nature of complex systems. It is also helpful in elucidating how the interaction between different types of shocks and crises creates entirely new, unpredictable phenomena with feedbacks and impacts that are difficult to anticipate (Westman et al., 2022).

As noted, CAS is also the theoretical foundation of CEF. Civic ecosystems are CAS composed of diverse and interdependent social actors and held together by shared civic values, which emerge and self‐organise to address specific social problems (Rangelov and Theros, 2023). Like other types of CAS, civic ecosystems involve multiple inhomogeneous agents that interact with each other and with the environment, and through these interactions learn and innovate in ways that enable them to adapt their behaviour and create system change.

In addition to diversity and interdependence, another key characteristic of civic ecosystems is civicness, which refers to a mode of behaviour driven by concern for the public interest and common good (Kaldor, 2019; Kaldor and Radice, 2022). While civicness can be observed in all societies, it is rooted in different secular, moral, religious, and spiritual traditions, and different logics and forms of social organisation that are culturally situated (Turkmani, 2024). Analytically, civicness helps to determine the composition and boundaries of civic ecosystems which often operate across civil society, the state, and the market, as well as across geographies, by directing attention to the behaviour of ecosystem constituents.

Civic ecosystems are distinguished from related concepts such as civic networks by their distinctive logic. Both ecosystems and networks have been analysed as CAS (Lansing, 2003), but as patterns of social relations addressing specific social problems, ecosystems and networks are quite different. The network logic is about connection and communication, captured by the density of ties between actors and the flows of information and communication; it is these flows that constitute the network. In contrast, the ecosystem logic is about diversity and interdependence, captured by the range of actors and the pathways through which they interact with one another and with their shared environment; the pathways constitute the ecosystem. While the study of networks underscores centrality, density, and degree of connection, network models do not necessarily imply adaptation, feedback, or co‐evolution among the participants. These are, though, critical in the study of ecosystems that shifts the emphasis to interdependencies and co‐evolution.

These differences have important implications. Analytically, complexity science and systems thinking are better lenses for understanding ecosystems than network analysis. Practically, networks are good at fostering collaboration whereas ecosystems excel at fostering complementarity. Civic ecosystems can generate complementarity through accommodation and symbiosis, as well as competition. Individual ecosystem constituents may be driven by competing logics of action and theories of change—principle versus pragmatism or top‐down versus bottom‐up—but collectively, they may be complementary and mutually reinforcing at the level of the ecosystem as a whole (Rangelov and Theros, 2023, p. 799).

CEF scholarship shows how complementarity can take different forms. For example, ‘niche complementarity’ encourages specialisation and increases the resources, ideas, and practices available within the ecosystem, whereas ‘scale complementarity’ expands the ecosystem by involving new actors and leveraging new capabilities for tackling the problem at stake (Rangelov and Theros, 2023, p. 803).

Another important insight from CEF scholarship is that certain properties of civic ecosystems can serve both to enable and constrain their adaptive responses to crises. The informality and agility of civic ecosystems allow them to fill gaps and adapt to dynamically changing contexts rapidly and effectively; however, they can also divert attention and resources from the development of long‐term solutions, coordination, and institutionalisation (Czerska‐Shaw and Dunin‐Wąsowicz, 2025). In compound crises, this means that learning and innovation may accelerate in moments of acute shock and disruption but slow down between them. This behaviour of civic ecosystems is consistent with findings in the literature on complex social‐ecological systems, which show how systems change through adaptive cycles of continuity, abrupt crisis, and reorganisation (Walker et al., 2020).

## 3 COMPOUND CRISES IN NORTHERN SYRIA

Understanding the context in which the February 2023 earthquakes struck northern Syria is essential for grasping both the impact of the disaster and the response effort. They affected a region already grappling with more than a decade of armed conflict, political fragmentation, economic collapse, geopolitical rivalry, and a protracted humanitarian emergency. The disaster unfolded in and added to these compound crises, where overlapping shocks and disruptions intersected to create an environment of extreme vulnerability. It exposed pre‐existing fractures within Syria's already fragile humanitarian and governance systems and highlighted the significant barriers to international humanitarian assistance delivery. These factors shaped the challenges and opportunities facing an array of local, transnational, and international actors who mobilised to provide relief.

### 3.1 Conflict and fragmentation in Syria

Since the start of the Syrian uprising in 2011, the country had been mired in a prolonged and multifaceted conflict characterised by territorial fragmentation and the presence of multiple competing groups and governance structures. By the time of the earthquakes, the Syrian regime, backed by Russia, Iran, and militias such as Hezbollah, had managed to reclaim large swaths of territory, including the country's major cities, such as Damascus and Aleppo, as well as much of the central, coastal, and southern areas, but had failed to reassert full control over the country. Opposition‐held areas, predominantly in the northwest, including Idlib and northern Aleppo, remained outside of regime control, governed by fragmented political and military structures. Meanwhile, the Kurdish‐led Syrian Democratic Forces (SDF) controlled the northeast, including parts of Hasakah, Raqqa, and Deir ez‐Zor, operating under a semi‐autonomous administration that faced geopolitical constraints both from the Syrian regime and Türkiye (EUAA, 2023). These divisions created a fragmented governance landscape, exacerbating humanitarian challenges and limiting the ability of institutions to respond to crises effectively (UNHCR, 2023).

The war had devastating consequences for Syrian society. It led to mass displacement, with millions of Syrians becoming refugees or internally displaced persons (IDPs). Infrastructure was severely degraded, and essential services, such as healthcare, education, and housing, collapsed in many parts of the country. The reliance of opposition‐held areas on international humanitarian aid was also a key issue, with cross‐border assistance subject to political negotiations at the United Nations (UN) Security Council. The Syrian regime sought to limit access to cross‐border aid, using its control over assistance as a political tool, further complicating efforts to address the humanitarian crisis (Human Rights Watch, 2023).

### 3.2 Northwest Syria: a region of overlapping and interlinking crises

Northwest Syria stands out as a region particularly affected by political fragmentation and humanitarian constraints, making it especially vulnerable to further shocks and disruption. At the time of the earthquakes, the region was governed by several competing entities: the Syrian Interim Government (SIG), which operated in Turkish‐influenced areas of northern Aleppo; and the Syrian Salvation Government (SSG), affiliated with Hay'at Tahrir al‐Sham (HTS), which exerted control over Idlib and adjoining parts of western Aleppo. These governance structures lacked full legitimacy and exhibited weak institutional capacity, making coordinated governance and disaster response particularly difficult (iMMAP FSL Unit et al., 2023; Alkhalil et al., 2024).

The northwest was also home to a large displaced population. An estimated 2.9 million IDPs lived in the region, including 1.9 million in makeshift camps. Even before the earthquakes, these communities faced severe vulnerabilities due to inadequate shelter, a lack of access to clean water and sanitation, and recurrent military escalations. The reliance of the region on Türkiye for humanitarian access and political backing further complicated the earthquake response, as Türkiye's own emergency priorities and its influence over opposition authorities affected the timing and movement of assistance; for example, aid from northeast Syria was reportedly delayed pending Turkish clearance (Jabbour et al., 2023).

In contrast, the SDF‐controlled northeast operated under a semi‐autonomous governance model, facing its own set of challenges. The area remained isolated owing to political tensions with both the Syrian regime and Türkiye, which imposed significant restrictions on aid access. This political isolation limited the region's ability to respond effectively to crises and receive international humanitarian support (Tokmajyan and Khaddour, 2022).

### 3.3 The earthquakes and their immediate impact

The earthquakes on 6 February 2023 affected northern Syria and southern Türkiye. They caused widespread destruction, particularly in Syria's northwest provinces, Aleppo and Idlib, which were heavily affected by ongoing armed conflict at the time. The disaster exacerbated an already dire humanitarian emergency by destroying homes, schools, hospitals, and roads, and displacing an additional 103,000 people (OCHA, 2023). More than 6,000 people lost their lives. IDPs living in fragile and poorly constructed shelters were among the hardest hit, with many camp structures collapsing under the impact of the earthquakes.

The immediate humanitarian response was severely constrained by geopolitical challenges. The region's reliance on cross‐border aid was complicated by a long‐standing blockade of cross‐line assistance imposed by the Syrian regime. The demands of Türkiye's own disaster response further limited its willingness and ability to facilitate aid flows into northwest Syria, intensifying the already severe delays caused by ongoing negotiations between the UN and the Syrian regime in the aftermath of the earthquakes. The UN's response was delayed and international aid failed to reach northwest Syria for nearly a week, largely because of bureaucratic and political hurdles at the Security Council. The eventual approval of additional aid crossings a week later on 13 February, provided some relief, but the temporary nature of the authorisation highlighted the precarious state of humanitarian access in the region (Barber, 2023; Human Rights Watch, 2023).

The earthquakes also exposed the lack of political will of the Syrian regime to facilitate humanitarian interventions, while revealing the complex and often conflicting dynamics of governance in that part of Syria. As the governance arm of the Islamist group HTS, the SSG's inability to coordinate a centralised disaster response underscored its weak institutional control, worsened by Türkiye's limited capacity to provide it with direct support in the aftermath of the disaster. Instead, the SSG sought to leverage the earthquake response to strengthen its legitimacy by controlling aid flows and asserting its dominance over humanitarian operations in Idlib (Hatahet, 2023).

The delayed international response led to widespread moral outrage and a sense of abandonment among affected communities. But it also spurred rapid mobilisation and action by an array of new and established humanitarian actors and informal networks at the local level, some of which crossed conflict lines, as well as within the Syrian diaspora. The scale and speed of mobilisation underscored the resurgence of civicness and the resilience of civic actors in the face of yet another abrupt crisis, even as formal structures faltered (al‐Jabassini and Ezzi, 2023). As one respondent noted: ‘The failure of the HTS and the SIG to provide governance capacity created space for health directorates, informal professional networks, and tribal structures to assert themselves’. Specifically, it strengthened the leadership role of the Idlib Health Directorate and Syrian Civil Defence (White Helmets), and their ability to network with the diaspora. The crisis also created opportunities for military factions to build trust and gain legitimacy through participation in the relief effort.

## 4 METHODS

This study combines concepts and ideas from CAS theory, CEF scholarship, and the compound crises literature to develop an analytical framework for assessing and interpreting data and evidence collected through key informant interviews (KIIs), a focus group discussion (FGD), and participatory observation. The analysis of the empirical evidence collected at different stages of the research informed an iterative refinement of the analytical framework.

Eighteen semi‐structured KIIs (n = 18) were conducted in two stages. Thirteen KIIs were carried out in the immediate aftermath of the earthquakes (February and March 2023) with respondents from local and international NGOs, international organisations, and local health authorities and facilities, as well as with community leaders and diaspora members. The interviews captured early data on the unfolding disaster response and how it interacted with the armed conflict, mass displacement, and humanitarian emergency already affecting the area.

The FGD was held in June 2024 with seven participants, comprising civil defence personnel, religious and tribal leaders, and staff of local and international NGOs and international organisations. It was structured in two parts: chronological and thematic. The goal was to track the timeline of the crisis response to gather data about the emergence, impact, and limitations of the civic ecosystem. The discussion focused on identifying patterns of behaviour among a range of civic and uncivic actors, and understanding their interactions, interdependencies, and complementarities.

A further five KIIs were carried out in March 2026 to fill gaps in evidence and to consider the impact on ecosystem properties and dynamics of the transition in Syria following the overthrow of President Bashar al‐Assad.

All KII and FGD data were collected with the informed consent of respondents, transcribed and translated from Arabic to English, anonymised entirely, and stored securely in an encrypted digital environment.

In addition, participatory observation provided important insights across our research cycle. The five Syrian co‐authors were directly involved in the earthquake relief efforts either on the ground or remotely, enabling a form of situated knowledge and reflexive inquiry that informed the analysis. The research process was shaped by ongoing dialogue among the co‐authors with lived experience of and direct observations from the aftermath of the disaster and those with analytical insights from civic ecosystem research in other crisis contexts. This iterative process allowed us to refine the analytical framework in a dynamic manner with emerging evidence.

The methodology draws on a growing body of scholarship on co‐produced knowledge, which emphasises how reflexivity, positionality, and collaboration within interdisciplinary teams shape nuanced and contextually grounded understanding (Jagosh et al., 2012; Fazey et al., 2020; Turnhout et al., 2020).

## 5 FINDINGS AND DISCUSSION: A CIVIC ECOSYSTEM FOR CRISIS RESPONSE

Our research revealed how a civic ecosystem for crisis response emerged and self‐organised in rapid order in the immediate aftermath of a disaster that compounded the multiple crises already affecting northern Syria. This section combines discussion of the collected data and evidence with ideas and insights from CEF scholarship and the CAS literature to analyse how that ecosystem emerged, to assess its impact, and to examine its limitations.

### 5.1 Emergence

The immediate disaster response was carried out by national and local NGOs, spontaneously emerging community‐led initiatives and informal networks. Despite facing resource constraints and suffering losses themselves, local responders swiftly mobilised to perform urgent relief operations, informed by their deep understanding of the socio‐political dynamics of the region and experience of other co‐occurring crises. Their efforts encompassed search and rescue operations, provision of emergency shelter, medical care, and collection and distribution of essential food and non‐food items to affected communities.

The mobilisation of local civic actors included government‐controlled territories. Individuals, informal civil society networks, and faith‐based organisations coordinated relief efforts in spite of security constraints. Many circumvented official state apparatuses to collect and channel aid across conflict lines. There was also rapid but discreetly pursued humanitarian mobilisation among civil servants and members of state‐run syndicates who, despite operating within regime‐controlled institutions, quietly raised funds and resources to support the disaster response in northwest Syria (al‐Jabassini and Ezzi, 2023). Parallel efforts emerged in the SDF‐controlled northeast, where civil society organisations (CSOs) and traditional tribal networks played a key role in circumventing the political isolation imposed by the Syrian regime and Türkiye. Responding to mounting pressure from CSOs and tribal leaders, these networks rapidly coordinated large‐scale aid convoys (Tokmajyan and Khaddour, 2022). Their ability to facilitate cross‐line assistance highlighted how informal actors can shape humanitarian responses outside of formal structures.

Within a few hours of the earthquakes, Syrian diaspora groups assumed an active role and started coordinating among themselves and with northwest Syria‐based local NGOs. They launched a large‐scale fundraising campaign, disseminated critical information using rapid needs assessments and satellite imagery, and lobbied international policymakers. An estimated total of USD 51.5 million was raised by Syrian diaspora groups in the first week (Agha, 2023).

The civic ecosystem for disaster response exhibited several key characteristics of CAS. Emergent behaviour—micro‐level interactions of ecosystem constituents influencing the behaviour of the whole system and resulting in system‐level patterns and properties that could not be predicted—produced a system‐wide spike in civicness. The resurgence of civicness as a central property of the system surprised some Syrians and observers. As one respondent put it: ‘The response appeared as if it were coordinated, and people knew their roles without guidance from any authority. This surprised everyone; even we were astonished by the speed and level of engagement’.

The civic mobilisation after the earthquakes, however, is consistent with disaster research and community resilience scholarship showing how acute crises can reveal or reactivate pre‐existing networks of solidarity, mutual aid, and informal organisation that are already embedded within communities (Dynes, 1970; Aldrich, 2012; Twigg and Mosel, 2017). The surge in civicness triggered by the earthquakes can be explained in part by the resilience of social systems under stress and the reactivation and reconfiguring of such networks. It is also consistent with CAS theory, which emphasises the tendency of decentralised agents to self‐organise and mobilise available resources in responding to shocks.

The devastation wrought by the earthquakes activated established civic actors, such as NGOs and health directorates, and spurred others into action, such as informal and diaspora networks. Their interplay in the ecosystem, in turn, created space and incentives for additional actors to join the relief effort. For example, Syrian contractors working in currency exchange in southern Türkiye and northern Syria played an important role in providing emergency relief. According to a respondent involved in these networks, they ‘formed a team called 9800 and provided a massive response in search and rescue operations, providing diesel, medicine, food, generators, and medical equipment maintenance. The contractors’ response began on the first day’.

Diaspora mobilisation generated new resources and created new tools that enabled an array of local actors to get involved on the ground and contributed to the spike in civicness. A health practitioner working in a diaspora‐funded medical organisation suggested that ‘the main office in the United States was the first to begin coordinating our organisation's staff in hospitals within Syria. They were very supportive, as they had not been victims of the earthquake like us, and we also benefited from the time difference’.

Context and context‐based responses played a central role in the emergence of the ecosystem. The compound crises context—an abrupt shock occurring in a situation of multiple protracted events—shaped the initial conditions of ecosystem emergence after the earthquakes and established path dependency for ecosystem response and impact. We discuss some of the context‐based responses that constrained the ecosystem and inhibited impact in the subsection on limitations below. However, consistent with CAS theory, we also observed how the context itself was altered in part by dynamic interactions among ecosystem constituents.

One example is how the behaviour and interactions of civic actors in the ecosystem shifted the calculations and behaviour of uncivic actors—armed groups and related authorities—by changing the opportunity structures for their participation. In the initial disaster response, some uncivic actors provided tacit or direct support to civic actors. Members of opposing factions temporarily set aside their political rivalries to support rescue and relief efforts. For instance, the HTS, partly seeking to gain local legitimacy, recognised its own lack of capacity to address the emergency and refrained from obstructing those who assumed leading roles. It provided security, machinery, and fuel, and benefited because the majority of HTS members and their families were from the affected area. This is consistent with other research showing how such pragmatic or solidaristic behaviour of armed groups reflects their social embeddedness within local communities and strategic adaptation to shifting incentives during crises (Arjona, 2016; Walch, 2018; Jackson, 2021).

Their tacit and direct support, in turn, expanded the space for others to join the relief effort. One respondent stated that ‘the local community response and individual volunteers nearly doubled the overall impact, as the number of volunteers involved in rescue operations was approximately equal to the number of civil defence personnel’. This is also an illustration of non‐linearity in the system: the behaviour and interactions of civic actors expanded the ecosystem, and expanded civic space, *via* their interplay with uncivic actors.

Context‐based responses were also rooted in long‐standing social norms, strong community ties, and the enduring power of collective action. In tribal communities, culturally embedded practices such as 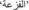 (*fazʿa)*—a tradition of communal support in times of crisis—played a central role in catalysing swift and effective action. A striking example was the dispatch of 80 relief trucks from northeast to northwest Syria, surpassing all other aid deliveries during the initial phase of the response. As one respondent explained: ‘There is a national and humanitarian dimension to the response of the tribes, but there is also the concept of *fazʿa* that characterises the people of the region’. Tribal solidarity was further reinforced by existing kinship ties, as many earthquake victims in the northwest were relatives of tribal communities in the northeast. It is also worth noting that the tribal aid convoys were particularly appreciated by local people since they included context‐sensitive non‐food items, such as typical Syrian soft furnishings and oil heaters that were far more responsive to their needs and considerate of their dignity than international aid.

This spirit of volunteerism was also evident in the medical field. Volunteer medical personnel—many of whom were off‐duty and themselves affected by the disaster—rushed to local hospitals to support overwhelmed healthcare teams. ‘In each hospital, there was a surgical medical staff equivalent to three times the original staff’, reported one of our respondents, underscoring the scale and dedication of civic mobilisation.

According to respondents, the spike in civicness was rooted in Islamic values and practices related to religious duty and spiritual accountability, as well as national identity and solidarity. For example, Syrian Civil Defence adopted a Quranic verse as its slogan, ‘whoever saves a life, it is as if they have saved all of humanity’, while mosques became important logistical hubs. One respondent pointed out that ‘Islamic forms of social organisation were more visible than purely religious or doctrinal motivations; it was more often described in terms of conscience, dignity, and Syrian solidarity’. Another observer cautioned against ‘overstating the Islamic framing of the response because many volunteers, especially in the diaspora, were motivated by their Syrian national identity and empathy as much as faith’.

### 5.2 Impact

The civic ecosystem that emerged in February 2023 was very effective in mounting a rapid, decentralised, and multifaceted response to the earthquakes, as both new and established actors rose to meet urgent needs on the ground. Its ability to self‐organise, scale, and mobilise complementary assets and capabilities swiftly, while circumventing structural constraints through creative, and at times atypical, forms of behaviour, was brought out by our research. Through their interactions and complementarities, ecosystem constituents were able to fill critical gaps, navigate barriers, and deliver life‐saving assistance in a situation where international humanitarian actors and fragmented public authorities failed.

When the earthquakes struck northern Syria, both the local population and Syrians in the diaspora were acutely aware that the de facto authorities in the affected areas had limited capacity to respond. That awareness shaped their behaviour and catalysed the ecosystem, which hastily self‐organised to address needs and fill gaps on the ground. In contrast, in the parts of Türkiye affected by the earthquakes, people expected the authorities to step in and provide relief, but that response proved to be inadequate. ‘Syrians in Türkiye took to the streets and waited for the state to respond, while we engaged in responding immediately because we realised that our authorities were not capable of responding to such a disaster’, stated a respondents based in southern Türkiye. These divergent expectations and modes of behaviour help to explain the contrasts between the early relief effort in Syria and in Türkiye, which surprised some observers. As one respondent put it: ‘People in northwest Syria know that there is no one to rescue them, and no one will respond to them, so there is no hope except for [relying on] themselves’.

The impact of the ecosystem stemmed in part from its ability to generate complementarity, which is the organising logic of civic ecosystems arising from the diversity and interdependence of their components (Rangelov and Theros, 2023). We observed two different types of complementarity at the level of the system: niche and scale. ‘Niche complementarity’ emerged spontaneously as diverse actors harnessed their distinctive experience, knowledge, capabilities, and connections to adapt and respond collectively. It involved specialisation, building on the pre‐existing strengths of established humanitarian actors and their experience of managing other co‐occurring crises. The White Helmets led rescue operations; MSF (Médecins Sans Frontières) Belgium provided mobile clinics; the Idlib Health Directorate organised the health response in the Idlib Governorate; the private sector provided crucial material support, such as diesel and power generators, to health facilities; and diaspora networks ran advocacy and fundraising campaigns and supplied technical support to local actors. We found that niche complementarity also involved atypical behaviour as some ecosystem actors assumed roles and functions that were very different from their usual interests and behaviour. In Turkish‐controlled areas, for instance, armed groups filled gaps in the capacity of the White Helmets and became integrated in their operations by providing manpower and machinery for the relief effort. In another case, we discovered that informal networks of jewellers approached hospitals to see what they needed and how they could help. They secured, for example, an electricity generator for one of the hospitals.

**FIGURE 1:**
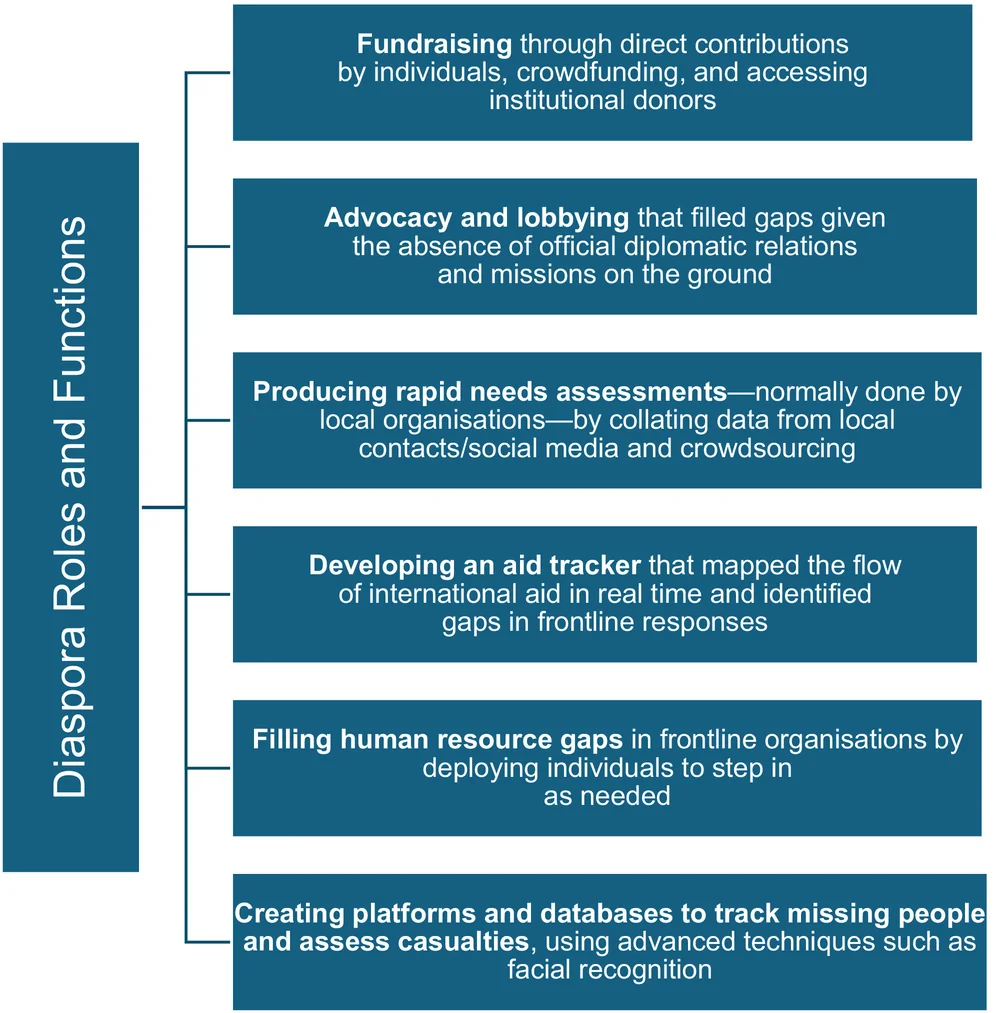
Civic ecosystem roles and functions of the Syrian diaspora. **Source:** authors.

We also detected the prevalence of ‘scale complementarity’, which involves a shift in the opportunity structures within the ecosystem that enables new actors to get involved and leverage additional capabilities and resources to address the problems at stake, thereby expanding the ecosystem as a whole (Rangelov and Theros, 2023). Figure 1 presents some of the main roles played by the diaspora in the relief effort in the immediate aftermath of the earthquakes.

Scale complementarity can be explained in part by the strong pre‐existing connectivity between individuals, networks, and institutions inside Syria and in the diaspora, which helped to facilitate the rapid expansion of the ecosystem in the wake of the earthquakes. In the health sector, for example, Syrian diaspora doctors' networks and organisations were already playing a key role in supporting and funding the sector before the disaster occurred; but the number of diaspora members who became involved after the earthquakes grew exponentially. According to our FGD participants, within a few hours of the earthquakes, Syrian diaspora groups started coordinating their efforts, among themselves and with northwest Syria‐based local NGOs, launching fundraising campaigns, disseminating critical information using rapid needs assessments and satellite images, and lobbying policymakers. A number of people immediately left for northwest Syria and southern Türkiye to help with the response operation, with some arriving on the ground within 24 hours. The evidence suggests that, in situations of compound crises, scale complementarity arises both through the activation of actors already involved in managing other co‐occurring crises and from rapid mobilisation of new actors, capabilities, and resources.

Complementarity also fostered innovations. Some of those generated by the civic ecosystem in the immediate post‐earthquake environment have not been sustained, but others have become consolidated and embedded in durable practices and institutions. Crowdfunding techniques developed and employed by the diaspora in the earthquake response have since been integrated into the fundraising strategies of many local organisations in Syria. This has given them greater flexibility to plan and carry out their operations and a degree of independence from international donors. It has also reinforced a sense of togetherness and pride among Syrians inside the country and in the diaspora, a feeling of being one community.

The impact of the ecosystem can also be explained by non‐linear feedback loops, which are a key characteristic of self‐organising complex systems (Meadows, 2008). For example, we uncovered a pattern that involved the disaggregation of social structures and institutions into separate components driven by different, and often competing, logics of action. These fractures enabled the civic ecosystem to overcome certain pre‐existing constraints and barriers that had arisen during the armed conflict and become embedded in specific institutions. The behaviour of the ecosystem could not be predicted from the behaviour of the institutions, which were largely unable to provide an adequate response to the disaster. At their margins, however, individuals came together in informal networks through which they were able to participate in the ecosystem and contribute to the relief effort.

We observed this dynamic playing out in regime‐controlled areas. For instance, trade unions in such areas were typically controlled by the regime and the Ba′ath Party, which meant that their members were unlikely to mobilise and take action without the approval of the authorities. But we discovered that members of a trade union in a regime‐controlled area had organised informally and delivered assistance to people affected by the earthquakes in areas not controlled by the regime. Some networks of civil servants had acted in similar ways. For example, civil servants and members of state‐run syndicates and technical directorates in regime‐controlled territories managed to collect discreetly financial contributions from their staff to alleviate the burden of NGOs in northwest Syria.

We also observed how the spike in civicness helped to transcend political‐military boundaries and enable the flow of goods and assistance across the conflict line separating northwest and northeast Syria. For instance, the tribal mobilisation in the northeast to provide aid for affected areas in the northwest, discussed in the previous subsection, was described by respondents as *Fazʿat Al‐Ashaer*, meaning ‘the rapid compassionate response of the tribes’. The Kurdish self‐administered authority was the second major source of support sent from northeast Syria to the northwest. These responses created positive feelings and further played into a sense of Syrian togetherness, breaking the long‐standing blockade of cross‐line aid operations between the SDF‐controlled northeast and the HTS‐controlled northwest.

Our findings suggest that in compound crises settings, a new and acute emergency can not only exacerbate pre‐existing risks and vulnerabilities but also create opportunities to reverse—at least partially and temporarily—some of the adverse consequences. The evidence also suggests that civic ecosystems can exploit abrupt crises to overcome some of the constraints and barriers associated with protracted crises.

The impact of the civic ecosystem was strongly influenced by its adaptive capacities. Adaptation occurred in response to a rapidly changing environment that presented ecosystem constituents with both challenges and opportunities. The barriers to delivering international humanitarian assistance, which we discuss below, spurred the ecosystem to mobilise other resources and develop alternatives. Yet, adaptation also occurred as a result of learning and ‘remembering’ from other shocks and disruptions associated with the ongoing armed conflict, mass displacement, and humanitarian emergency. A member of the Idlib Health Directorate who participated in the FGD emphasised that the institution's ability to respond rapidly, without waiting for orders or written plans, was rooted in tacit knowledge developed over years of conflict. In previous moments of acute crisis, such as the COVID‐19 pandemic, health directorates established WhatsApp groups to coordinate their efforts in local areas. Each subsequent crisis was used to expand and update the lists they had developed of key individuals and groups that could provide relief. When the earthquakes struck, this enabled the Idlib Health Directorate, for example, to create quickly WhatsApp groups for different needs and purposes in each affected area. In other words, in compound crises settings, civic ecosystems evolve through adaptive cycles of learning and ‘remembering’ across multiple co‐occurring crises.

### 5.3 Limitations

Some of the limitations of the civic ecosystem response to the earthquakes in northern Syria can be explained by the influence of the compound crises context in which the disaster occurred. The ongoing protracted crises already affecting the area shaped the initial conditions for the emergence of the ecosystem and created path dependencies that constrained its functioning and effectiveness in several ways. The earthquakes devastated the limited critical infrastructure and governance capacity in the area, which had already been depleted by the long‐standing armed conflict and humanitarian emergency. Civic actors managed to fill some of the gaps and integrate into the ecosystem some governance structures of the de facto authorities in different parts of northwest Syria. However, these structures had few capabilities and resources to contribute to the relief effort, affecting the overall ecosystem response.

The evidence shows how such limitations played out across different authorities and areas, including the SIG in Turkish‐influenced areas of northern Aleppo, the SSG affiliated to HTS in Idlib and adjoining parts of western Aleppo, and the regime‐controlled area of Aleppo city. As one of our interviewees pointed out: ‘One of the reasons why SIG's Ministry of Health did not respond in the aftermath of the earthquake is because they have nothing to offer: no warehouses, ambulances, or human resources’. In Idlib, interviewees suggested that the SSG's response was marginally better, but still, ‘they did not provide anything tangible from their Ministry of Health’. Another respondent underlined that ‘the Syrian regime did not have the capacity to respond, nor the desire to allow civil society organisations to respond. It feared the erosion of its weak legitimacy and the emergence of community leaders outside its authority’.

The dynamic interactions between co‐evolving ecosystem components and between the ecosystem and its highly volatile and dynamic environment account for another set of system‐wide limitations. The interactions between the international and local components of the ecosystem, for example, were significantly hindered by several events. One was the decision of the Turkish government to close the Turkish‐Syrian border immediately after the earthquakes ostensibly due to logistical problems and damaged roads leading to the crossing point of Bab al‐Hawa; however, Türkiye probably wanted to focus on its own population affected by the disaster (Hilani, 2023). Closure of the Bab al‐Hawa checkpoint and delays in opening additional humanitarian crossings significantly impeded the flow of resources into Syria, exacerbated local shortages, and prevented UN agencies based in south Türkiye from delivering urgently needed humanitarian aid in the first week (Alkhalil et al., 2023). Another factor was the long‐standing blockade of cross‐line humanitarian assistance imposed by the Syrian regime and the reluctance of the UN to expand its mandated cross‐border operations without consent from the regime in Damascus.

Our research also revealed an inability of the civic ecosystem to sustain some of the gains made in the early earthquake response. The cooperation of armed actors was rather short‐lived and pre‐existing patterns of behaviour quickly reemerged. The HTS, for example, reasserted its control and, especially after IDP camps were established, reverted back to interfering and impeding operations. Similarly, in Turkish‐controlled areas, local armed groups pivoted back to their Turkish sponsors. Long‐standing Arab‐Kurdish tensions and the perception of politicised aid in Kurdish‐led support to affected areas reversed some of the positive gains in intra‐community relations enabled by that assistance, although this was more salient in Turkish‐controlled areas. This pattern is consistent with CAS theory, which predicts shocks triggering short‐lived phases of reorganisation before the system settles back and old behaviours return.

Yet, some of the gains made by the ecosystem in the aftermath of the earthquakes ended up changing the context itself, even if they did not alter the overall behaviour of armed groups in the long term. Because civic actors took the initiative and asserted themselves in the disaster response, they gained legitimacy, respect, and connections with some members of the armed groups and de facto authorities. Our evidence reveals that civic space emerged or expanded for a range of actors, including those in quasi‐governmental institutions such as the Idlib Health Directorate. That aspect of the ecosystem response was more evident in the policies and procedures of governance structures, especially the health directorates—exhibited also in earlier responses to COVID‐19—in comparison to other actors.

Some of the limitations of the ecosystem have to do with the nature of civic ecosystems as particular kinds of social relations. Consistent with insights from CEF scholarship, the agility and informality of the ecosystem, which shaped its ability to respond rapidly and effectively, also diverted attention and resources from developing long‐term solutions and sustaining innovations (Czerska‐Shaw and Dunin‐Wąsowicz, 2025). For instance, the inability to develop and institutionalise coordination mechanisms that could build on the constituents and contributions of the ecosystem has limited long‐term impact. As one respondent stated: ‘Coordination among stakeholders was excellent, but declined significantly after the acute response’.

However, our research on the impact of the collapse of the regime in Damascus and the emergence of the HTS as the transitional government complicates this analysis. Some of the learning and innovation that had been generated by Syria's protracted crises and reinforced by the earthquake response have been scaled and institutionalised in the post‐Assad period. For example, Syrian Civil Defence directorates and personnel have been effectively transformed into the newly established Ministry of Emergency and Disaster Management in the transitional government. Most of the leaders in the Ministry of Health are individuals who previously worked in the Idlib Health Directorate and health NGOs that responded to the multiple crises and shocks in northern Syria. In other words, in compound crises settings, civic ecosystems can ‘store’ learning and innovation generated from both protracted and acute crises. When the environment shifts and opportunities arise, learning and innovation can be ‘retrieved’ by the ecosystem and institutionalised at scale. One question for further research is how this might play out in Syria given concerns about the exclusionary nature of the transitional government and uncertainty about the long‐term trajectory of the country.

## 6 CONCLUSIONS AND IMPLICATIONS

Compound crises present distinctive challenges and opportunities for addressing them, which are not sufficiently understood. They tend to amplify vulnerabilities and exacerbate structural constraints and barriers to effective responses, which are the main focus of the literature on compound crises. Our findings, however, suggest that the co‐occurrence of multiple crises can also change the context or environment by creating new capacities and shifting social relations in productive ways. In particular, abrupt shocks can create opportunities for tackling some of the entrenched impacts of protracted crises.

Civic ecosystems are particularly adept at exploiting such opportunities. One conclusion that emerges from the study is that in compound crises settings, civic ecosystem responses to a sudden shock can build on experiences from, and shift some of the constraints and barriers generated by, protracted crises. In other words, civic ecosystems evolve through adaptive cycles of learning and innovation across multiple co‐occurring crises.

Moreover, learning and innovation that may appear lost are in fact stored in the ecosystem. They can be retrieved to address future challenges, such as responding to further shocks and disruptions, or to exploit emerging opportunities, such as pursuing institutionalisation when favourable changes in the environment occur.

For crisis response practitioners and policymakers, one implication of this research is that a civic ecosystems lens makes visible hidden resources, capacities, and capabilities that can be activated and leveraged in addressing compound crises. While needs assessments are rightly emphasised by humanitarian actors, a civic ecosystem perspective provides an opportunity to generate asset maps and assessments that can be matched with needs as they arise and evolve on the ground.

An implication for scholars is that combining CAS theory and CEF scholarship has significant potential to advance our understanding and analysis of complex social problems. So far, social scientists have tended to harness the insights of systems theory and thinking to study how systems generate such problems, while leaving largely untapped their potential to understand how systems can also address them.

Another avenue for further research is the long‐term impact and institutionalisation of the civic ecosystem in northern Syria. The fall of the Assad regime and the emergence of the HTS as the leader of the transitional authorities in the country present an opportunity to track how learning and innovation generated by the ecosystem may shape the new constellation in unforeseen ways.

## FUNDING STATEMENT

The study was carried out under the framework of the Peace and Conflict Resolution Evidence Platform (PeaceRep) funded by the United Kingdom government's Foreign, Commonwealth & Development Office. The views expressed are those of the authors and do not necessarily reflect the official policy or positions of the UK government.

## ACKNOWLEDGEMENTS

We are grateful to our key informants and focus group participants inside and outside Syria, for their time and insights, and to anonymous reviewers for their deep engagement with the study and most helpful comments.

## CONFLICT OF INTEREST STATEMENT

The authors declare that there are no conflicts of interest with respect to the research, authorship, and/or publication of this article.

## Data Availability

The data that support the findings of this study are available on request from the corresponding author. The data are not publicly available due to privacy or ethical restrictions.
